# Alterations in the complement cascade in post-traumatic stress disorder

**DOI:** 10.1186/1710-1492-6-3

**Published:** 2010-02-21

**Authors:** Lilit P Hovhannisyan, Gohar M Mkrtchyan, Samvel H Sukiasian, Anna S Boyajyan

**Affiliations:** 1 Institute of Molecular Biology of the National Academy of Sciences of Armenia; 2 Stress Centre of the Ministry of Labour and Social Affairs, Armenia

## Abstract

**Background:**

In the present study we assessed the functional state of the major mediator of the immune response, the complement system, in post-traumatic stress disorder (PTSD).

**Methods:**

Thirty one PTSD patients within 13 years from traumatic event and the same number of sex- and age-matched healthy volunteers were involved in this study. In the blood serum of the study subjects hemolytic activities of the classical and alternative complement pathways, as well as the activities of the individual complement components have been measured. Correlation analysis between all measured parameters was also performed.

**Results:**

According to the results obtained PTSD is characterized by hyperactivation of the complement classical pathway, hypoactivation of the complement alternative pathway and overactivation of the terminal pathway.

**Conclusions:**

The results obtained provide further evidence on the involvement of the inflammatory component in pathogenesis of PTSD.

## Background

Post-traumatic stress disorder (PTSD) is a complex, severe and chronic psychiatric illness, an anxiety disorder (DSM-IV-TR code: 309.81; ICD-10 code: F43.1, F62.0) that can develop in a person after exposure to a terrifying event (or after witnessing or learning about such an event) or ordeal in which grave physical harm occurred or was threatened. Traumatic events that may trigger PTSD include violent personal assaults, natural or human-caused disasters, terrorist attacks, accidents, or military combats. The person's response to the event must involve intense fear, helplessness, or horror. PTSD is clinically manifested with three main syndromes: re-experiencing; avoidance behavior and numbing of emotion; and physiological hyperarousal, accompanied by a number of "somatic" pathologies. Symptoms usually begin within the first 3 months after the traumatic event and last for many years, although there may be a delay of months, or even years, before symptoms appear.

Patients with PTSD have a reduced quality of life, an increased number of suicides and hospitalizations, high frequency of depressions and alcohol drug abuse; social, family life and work become impossible [1-5].

The molecular pathomechanisms of PTSD are not well defined and only beginning to be understood, which has limited the progress in development of the efficient measures of PTSD-therapy. Promising findings suggest that both environment and genetic factors are involved in PTSD-generation mechanisms [6], and that alterations in the immune reactivity might be in a sufficient degree responsible for disease progression [7,8]. Moreover, there is a growing body of evidence on the important role of inflammation in pathogenesis of PTSD [9-17]. However, due to insufficiency of relevant data, a molecular picture of the immune system abnormalities in PTSD is yet unclear.

The complement system is major effector of the immune response, which acts on the interface of innate and adaptive immunity, and is a key component and trigger of many immunoregulatory mechanisms. Activation of the complement through classical, alternative or lectin pathways generates opsonins, anaphylatoxins, and chemotaxins, mediators of inflammation and apoptosis [18-20]. Alterations in the functional activity of the complement cascade contribute to the pathology of many human diseases [21-23], including mental disorders [24-32], and are also detected during physiological stress [33,34]. The alterations in the complement cascade have been considered as indicator of the implication of inflammatory component in disease etiology, pathogenesis and/or progression [21-23]. Whereas PTSD-affected subjects showed a low-grade systemic proinflammatory state, which, moreover, was related to PTSD symptom levels [9-17], the state of the complement system in PTSD have been never studied.

The aim of the present study was to assess the functional activity of the complement cascade in PTSD by determining total hemolytic activities of its classical and alternative pathways, and hemolytic activities of its individual components, C2, C3, C4, factor B and factor D, in the blood serum of PTSD affected and healthy subjects. C2 and C4 are main components of the classical pathway, factor B and factor D are essential components of the alternative pathway, and C3 is the initial point for the alternative pathway and a converge point of all three complement activation pathways, starting up for the terminal pathway [18-20]. In addition, correlation study between all measured parameters was also performed.

## Methods

### Study subjects

In the present study 31 PTSD affected subjects (males 27, females 4; mean age 42 ± 4.6 (mean ± S.E.)), combat veterans within 13 years from traumatic event were examined. All the affected subjects were hospitalized at the Stress Center. Blood sampling was performed before any medication was applied. Diagnosis of PTSD was determined by the Structured Clinical Interview for Diagnostic and Statistical Manual of Mental Disorders (DSM-IV (SCID-I) [35] and the Clinician Administered PTSD Scale (CAPS) [36]. Age- and sex matched healthy controls (n = 31) were volunteers from the Institute of Molecular Biology without any history of physical or sexual abuse or other major trauma, defined as being free of current or past psychiatric disorders as determined by structured interview (SCID-I) and leading an active and independent life. The exclusion criteria were participant reports on a) immune, cardiovascular, cerebrovascular, metabolic, oncological, or kidney disorders; b) current cold, respiratory or other infections; c) prescribed medication, use of any anti-inflammatory or antihistamine medication or immunosupressors in the last 12 months; c) any surgical invention in the last 12 months.

All subjects gave their informed consents to provide 5 ml of venous blood for the study, and the study was approved by the Ethical Committee of the Institute of Molecular Biology.

### Collection of blood and preparation of serum

Practically fasting blood samples were collected by venipuncture in appropriate tubes at 9:00-10:00 a.m. and kept on ice for 60 min. After that the blood was centrifuged at 3000 g for 15 min at 4°C to separate serum from blood corpuscles. The obtained serum samples were stored in aliquots at -30°C and thawed immediately prior to use. To check inter-test repeatability of the results, each study subject was sampled twice within the interval of 2 days. Healthy subject were additionally sampled after 6 months. That was impossible to perform in case of PTSD-affected subjects, because after the second sampling they have started to use medication and even after discharge from the hospital continued to use supporting therapy, which may interfere the results of this study.

### Hemolytic assays

Hemolytic activities of the complement classical and alternative pathways (CH50 and AH50, respectively) and of the complement components C2 (C2H50), C3 (C3H50), C4 (C4H50), factor B (fBH50), and factor D (fDH50) in the blood serum of PTSD-affected and healthy subjects were measured by earlier developed assays [37]. Measuring AH50, fDH50, and fBH50, rabbit erythrocytes were used as target cells. For CH50, C2H50, C3H50 and C4H50 assays, sheep erythrocytes sensitized with rabbit anti-sheep erythrocyte antibodies were used as target cells. The hemolytic activity was expressed in units/ml. One unit of hemolytic activity is defined as an amount of serum that causes a 50% hemolysis of erythrocytes in a reaction mixture. The hemolytic titer is the number of units per ml of serum, and is calculated as the reciprocal of the serum dilution, which gives 50% cell lysis. Sheep erythrocytes sensitized with rabbit anti-sheep erythrocyte antibodies (5 × 10^8 ^cells/ml) and rabbit erythrocytes (1 × 10^8 ^cells/ml) were prepared as previously described [38].

### Preparation of depleted sera

Factor B-, factor D-, C2-, C3- and C4-depleted sera for fBH50, fDH50, C2H50, C3H50 and C4H50 assays, respectively, were prepared according to previously developed methods [39]. Factor B- and C2-depleted sera were obtained by incubating human fresh serum in 50°C water bath for 20 min and 56°C water bath for 6 min, respectively. Factor D was selectively depleted from human serum by Sephadex G-75 gel filtration. C3 depleted serum was obtained by treatment of guinea pig serum with zymosan, and complement C4 depleted serum was obtained by incubation of guinea pig serum with 150 mM NH_4_OH for 45 min at 37°C. The efficiency of depletion (≥ 95%) was judged by ELISA.

### Statistical analysis

Data were analysed by Student's unpaired two-tailed t-test and Pearson's correlation analysis. A value of p < 0.05 was considered significant.

## Results

To assess the functional state of the complement in PTSD, we measured CH50, AH50, C2H50, C3H50, C4H50, fBH50, and fDH50 in the blood serum of PTSD affected and healthy subjects. The results obtained not depend on the period of blood sampling, thus demonstrating good inter-test repeatability, and are presented in table 1.

**Table 1:** Mean values of measured parameters in PTSD patients and healthy subjects.

Parameter	HS (M ± S.E.)	PTSD (M ± S.E.)	difference	p =
CH50	176 ± 24.56	375 ± 29.52	2.1↑	0.0002

C2H50	58.8 ± 3.1	67.6 ± 1.63	1.2↑	0.05

C3H50	55.92 ± 1.82	37.57 ± 4.2	1.5↓	0.002

C4H50	36.64 ± 7.68	60.1 ± 7.3	1.6↑	0.03

AH50	87.6 ± 2.13	52.3 ± 3.37	1.7↓	0.0001

fBH50	65.2 ± 12.9	40.8 ± 3.6	1.6↓	0.02

fDH50	163.7 ± 24.95	71.7 ± 3.99	2.3↓	0.001

According to the results obtained, mean values of serum CH50, C2H50 and C4H50 in PTSD patients were 2.1, 1.2 and 1.6 times significantly higher than in case of healthy subjects. On the contrary, mean values of serum C3H50, AH50, fBH50, and fDH50 in PTSD patients were 1.5, 1.7, 1.6, and 2.3 times significantly lower as compared to healthy subjects. The detected changes positively and significantly correlated (p < 0.05) with total (frequency and intensity) PTSD symptom cluster of re-experiencing, avoidance, and hyperarousal, and with PTSD total symptom score.

Correlation analysis also demonstrated that in PTSD affected subjects C3H50 is significantly correlated with C2H50 and C4H50 (r = 0.72; p = 0.002; r = 0.5; p = 0.05 respectively), and AH50 is significantly correlated with C3H50 (r = 0.57; p = 0.027). However, we did not observe other significant correlations among measured parametrs in PTSD. The results of correlation analysis are presented in table 2.

**Table 2:** Analysis of correlation between measured parameters in PTSD patients.

	r	p =
CH50 versus C2H50	0.108	0.7

CH50 versus C3H50	-0.24	0.39

CH50 versus C4H50	-0.42	0.12

C2H50 versus C3H50	0.72	0.002

C2H50 versus C4H50	0.12	0.66

C3H50 versus C4H50	0.5	0.05

AH50 versus fBH50	0.155	0.6

AH50 versus fDH50	0.21	0.44

fBH50 versus fDH50	0.17	0.52

AH50 versus C3H50	0.57	0.027

AH50 versus CH50	0.087	0.66

fDH50 versus C3H50	0.34	0.22

fBH50 versus C3H50	-0.44	0.1

No significant correlation between the above-mentioned parametrs was detected in the healthy subjects group (p > 0.05).

## Discussion

The complement system with its central position in innate and adaptive immunity mediates a variety of effector functions. It consists of more than 30 circulating proteins and a similar number of cell surface receptor and regulator proteins. It is a complex cascade involving proteolytic cleavage of serum glycoproteins often activated by cell receptors. This cascade ultimately results in induction of the antibody responses, inflammation, phagocyte chemotaxis, and opsonization of apoptotic and necrotic cells, facilitating their recognition, clearance, and lysis. Complement exhibits three activation pathways - classical, alternative, and lectin, initiated via separate mechanisms, and a single terminal pathway that results in a formation of the membrane attack complex and subsequent cell lysis [18-20].

During the past decades it has become evident that dysfunction of complement contributes to the pathology of many human diseases [21-23], including mental disorders (schizophrenia, Alzheimer's disease, Huntington's and Pick's diseases) [24-32], and is also detected during physiological stress [33,34]. However, no data on the state of the complement system in PTSD have been reported, whereas a number of studies suggest that a direct pathophysiological consequence of PTSD is chronic low grade activation of systemic vascular inflammation [15-17]. Compared to controls, patients with PTSD showed higher WBC count [39], circulating levels of C-reactive protein [13], interleukin (IL)-1b [14,9], IL-6 and IL-6 receptor [10,11], as well as lower levels of the anti-inflammatory cytokine IL-4 [12].

This study was focused on the functional state of the major mediator of the inflammation, the complement system, in PTSD. The results obtained clearly demonstrated that pathogenesis of PTSD is characterized by complement dysfunction including hyperactivation state of the complement classical pathway and hypoactivation state of the complement alternative pathway.

The alternative pathway of complement is activated following spontaneous hydrolysis of the thioester bond of native C3, resulting into binding of factor B, which is cleaved by factor D, generating the efficient alternative pathway C3 convertase C3bBb. Multifunctional complement protein C3 is the initial point of the alternative pathway, and, at the same time, a converge point of all three complement activation pathways, i.e. starting point for the terminal pathway [16-18].

Hypoactivation state of the alternative pathway together with decreased activity of the complement C3 component, detected in PTSD affected subjects, probably reflects depletion of the C3 component due to its overutilization through the terminal pathway. This suggestion is convenient with correlation data indicating positive correlation between CH50 and C3H50 and absence of any correlation between AH50 and fBH50, and AH50 and fDH50 in PTSD affected subjects. Thus, it is obvious that the alternative pathway in PTSD is supressed on the initial stage of its activation, and that PTSD is also characterised by overactivated terminal complement pathway. On the other hand, absence of correlation between AH50 and CH50 suggests that alterations in activities of the classical and the alternative complement pathways in PTSD are not interdependent. As it was mentioned above, alterations in the complement cascade have been considered as indicator of the implication of inflammatory component in disease etiology, pathogenesis and/or progression [21-23].

Our study demonstrates that PTSD is associated with dysfunction of the complement system, and reveals the altered chains of the complement cascade. The results obtained provide further evidence on the involvement of the inflammatory component in pathogenesis of PTSD demonstrated in a number of studies [9-17,39]. Here we hypothesize that neuroendocrine mechanisms related to PTSD [40,41] modulating the immune function [42,43] might affect the initial steps in the inflammatory cascade and thus influence alterations in the functional activity of the major mediator of the inflammatory response, the complement system. However, to address molecular mechanisms responsible for the development of inflammatory processes and complement dysfunction in PTSD as well as their role in PTSD pathogenesis further studies are needed.

## Conclusions

1. Pathogenesis of PTSD is associated with the complement system dysfunction, including hyperactivation state of the complement classical pathway, hypoactivation state of the complement alternative pathway and overactivation of the complement terminal pathway;

2. Alterations in the activities of the classical and the alternative complement pathways in PTSD are not interdependent;

3. The alternative pathway in PTSD is suppressed on the initial stage of its activation.

## Competing interests

The authors declare that they have no competing interests.

## Authors' contributions

LH carried out the collection of blood, preparation of serum samples, and performed hemolitic assays. GM participated in methodological design and coordination of the study participants, performed the statistical analysis and the interpretation of data and drafting of manuscript. SS was responsible for selection and diagnostics of PTSD patients, and organization of intervies with diseased and healthy subjects. AB generated the idea of the study, performed general supervision of the research works, and developed final version to be published. All authors read and approved the final manuscript.
